# Draft Genome Sequences of Violacein-Producing Duganella sp. Isolates from a Waterway in Eastern Pennsylvania

**DOI:** 10.1128/MRA.01196-18

**Published:** 2018-09-27

**Authors:** Regina Lamendella, Brooke A. Jude

**Affiliations:** a Department of Biology, Juniata College, Huntingdon, Pennsylvania, USA; b Reem-Kayden Center for Science and Computation, Program of Biology, Bard College, Annandale-on-Hudson, New York, USA; Georgia Institute of Technology

## Abstract

Five Duganella sp. bacterial isolates that synthesize violacein were cultured from a central Pennsylvania waterway. Violacein has antimicrobial potential, including chytrid-killing effects, relevant to amphibian declines worldwide. Whole-genome analysis of these five microbial isolates may provide insights to better protect amphibian communities from fungal infections using bioremediation.

## Abstract (precis)

Five Duganella sp. bacterial isolates that synthesize violacein were cultured from a central Pennsylvania waterway.

## ANNOUNCEMENT

Water samples were obtained from streams in eastern Pennsylvania, along the Marcellus Shale formation, where salamanders have been impacted by Batrachochytrium dendrobatidis (1). B. dendrobatidis can cause chytrid infections and contributes to the decline in worldwide amphibian populations. Bacterial strains BJB475, BJB476, BJB480, BJB488, and BJB489 were isolated by plating a single water sample (150 to 200 µl) from Crooked Run in North Union Township, Pennsylvania, on Reasoner’s 2A (R2A) agar and incubating at 22 to 25°C for 48 h. Five violet-pigmented colonies were subcultured for genomic analysis.

Genomic DNA extraction was completed with the Gentra Puregene yeast/bacteria kit (Qiagen) following the manufacturer’s protocol. Library preparation was performed using Illumina’s Nextera XT library preparation kit. The multiplexed, paired-end Illumina libraries (150 bp) were run using HiSeq sequencing technology on the Illumina HiSeq 4000 instrument. Data were then demultiplexed by sample, and raw data were sent for analysis (Wright Labs, Huntington, PA). Reads were assembled using a previously published local pipeline (2–4). Sequences were quality filtered using BBDuk from the BBMap package version 37.50, maintaining a Q-score cutoff of 10 (https://sourceforge.net/projects/bbmap). A draft whole-genome assembly was built using SPAdes version 3.11.0 (5) with *k*-mer sizes of 21, 33, 55, 77, 99, and 127. Contigs shorter than 500 bp, or consisting of fewer than four reads, were filtered out of the assembly.

Draft whole-genome assemblies of the five strains averaged 40.6 contigs, with a high of 48 (BJB489) and a low of 35 (BJB475) (Table 1). The average *N*_50_ value for all five assemblies was 576,048 bp (Table 1). The average genome size is predicted to be 7.207 Mb, with an average G+C content of 64.358% (Table 1), comparable to the OxaII cluster of Duganella previously described (6). The three genomes of BJB480, BJB488, and BJB489 are nearly identical in length and G+C content and are likely closely related or clonal isolates.

**TABLE 1:** GenBank accession numbers of isolates from water from Crooked Run in North Union Township, Pennsylvania

Isolate	No. of contigs	Genome size (Mb)	G+C content (%)	*N*_50_ (bp)	Median read depth (×)	Avg no. of CDS	GenBank accession no.
BJB475	40	7.0	62.92	483,567	545	6,234	QVIP00000000
BJB476	35	7.23	63.88	725,652	274	6,284	QVIO00000000
BJB480	37	7.268	65	655,410	925	6,400	QVIN00000000
BJB488	43	7.268	65	469,376	334	6,389	QVIM00000000
BJB489	48	7.2695	64.99	546,234	394	6,401	QVIL00000000

Assembled contigs were annotated using three methods, a local pipeline running Prokka (7), RASTtk, via the PATRIC pipeline (8, 9), and the NCBI Prokaryotic Genome Annotation Pipeline (PGAP) (10). BLAST search results for fragments of 16S rRNA for all five isolates were 99 to 100% identical to those of other Duganella species, specifically HH01 (6), and RAxML analysis further clustered these isolates with those in the earlier study (6). Annotations across the three annotation platforms yielded an average of 6,292 coding DNA sequences (CDS), with a high of 6,401 (BJB489) and a low of 6,234 (BJB475). As expected, the violacein biosynthetic operon (*vioABCDE*) was present in all annotations for all strains. Additionally, all genomes contained genes involved in swarming and gliding motility, as well as biofilm production, correlating with the growth phenotypes observed on solid agar growth medium.

Future work may reveal if different phylogenetic groupings of violacein-producing strains provide unique phenotypic benefits when colonizing particular environments. Research into native violacein-producing strains may also suggest optimal bioremediation strain candidates for amphibians, should chytrid infections worsen in this watershed.

### Data availability.

The whole-genome sequences have been deposited at DDBJ/ENA/GenBank (Table 1). The bacterial strain genome sequences described in this paper include QVIP00000000 (BJB475), QVIO00000000 (BJB476), QVIN00000000 (BJB480), QVIM00000000 (BJB488), and QVIL00000000 (BJB489).

## References

[1] Raffel TR, Michel PJ, Sites EW, Rohr JR 2010 What drives chytrid infections in newt populations? Associations with substrate, temperature, and shade. Ecohealth 7:526–536. doi:10.1007/s10393-010-0358-2. 21125308

[2] O’Brien K, Perron GG, Jude BA 2018 Draft genome sequence of a red-pigmented *Janthinobacterium* sp. native to the Hudson Valley watershed. Genome Announc 6:6635. doi:10.1128/genomeA.01429-17. PMC5754502 29301893

[3] Doing G, Perron GG, Jude BA 2018 Draft genome sequence of a violacein-producing *Iodobacter* sp. from the Hudson Valley watershed. Genome Announc 6:1422. doi:10.1128/genomeA.01428-17. PMC5754501 29301892

[4] Bettina AM, Doing G, O’Brien K, Perron GG, Jude BA 2018 Draft genome sequences of phenotypically distinct *Janthinobacterium* sp. isolates cultured from the Hudson Valley watershed. Genome Announc 6:70. doi:10.1128/genomeA.01426-17. PMC5773719 29348334

[5] Bankevich A, Nurk S, Antipov D, Gurevich AA, Dvorkin M, Kulikov AS, Lesin VM, Nikolenko SI, Pham S, Prjibelski AD, Pyshkin AV, Sirotkin AV, Vyahhi N, Tesler G, Alekseyev MA, Pevzner PA 2012 SPAdes: a new genome assembly algorithm and its applications to single-cell sequencing. J Comput Biol 19:455–477. doi:10.1089/cmb.2012.0021. 22506599 PMC3342519

[6] Haack FS, Poehlein A, Kröger C, Voigt CA, Piepenbring M, Bode HB, Daniel R, Schäfer W, Streit WR 2016 Molecular keys to the *Janthinobacterium* and *Duganella* spp. interaction with the plant pathogen *Fusarium graminearum*. Front Microbiol 7:11905–11917. doi:10.3389/fmicb.2016.01668. PMC5080296 27833590

[7] Seemann T 2014 Prokka: rapid prokaryotic genome annotation. Bioinformatics 30:2068–2069. doi:10.1093/bioinformatics/btu153. 24642063

[8] Wattam AR, Abraham D, Dalay O, Disz TL, Driscoll T, Gabbard JL, Gillespie JJ, Gough R, Hix D, Kenyon R, Machi D, Mao C, Nordberg EK, Olson R, Overbeek R, Pusch GD, Shukla M, Schulman J, Stevens RL, Sullivan DE, Vonstein V, Warren A, Will R, Wilson MJC, Yoo HS, Zhang C, Zhang Y, Sobral BW 2014 PATRIC, the bacterial bioinformatics database and analysis resource. Nucleic Acids Res 42:D581–D591. doi:10.1093/nar/gkt1099. 24225323 PMC3965095

[9] Wattam AR, Davis JJ, Assaf R, Boisvert S, Brettin T, Bun C, Conrad N, Dietrich EM, Disz T, Gabbard JL, Gerdes S, Henry CS, Kenyon RW, Machi D, Mao C, Nordberg EK, Olsen GJ, Murphy-Olson DE, Olson R, Overbeek R, Parrello B, Pusch GD, Shukla M, Vonstein V, Warren A, Xia F, Yoo H, Stevens RL 2017 Improvements to PATRIC, the all-bacterial Bioinformatics Database and Analysis Resource Center. Nucleic Acids Res 45:D535–D542. doi:10.1093/nar/gkw1017. 27899627 PMC5210524

[10] Tatusova T, DiCuccio M, Badretdin A, Chetvernin V, Nawrocki EP, Zaslavsky L, Lomsadze A, Pruitt KD, Borodovsky M, Ostell J 2016 NCBI Prokaryotic Genome Annotation Pipeline. Nucleic Acids Res 44:6614–6624. doi:10.1093/nar/gkw569. 27342282 PMC5001611

